# RETRACTED: Kim et al. Protection against Oxidative Stress-Induced Apoptosis by Fermented Sea Tangle (*Laminaria japonica* Aresch) in Osteoblastic MC3T3-E1 Cells through Activation of Nrf2 Signaling Pathway. *Foods* 2021, *10*, 2807

**DOI:** 10.3390/foods14213658

**Published:** 2025-10-27

**Authors:** So Young Kim, Hee-Jae Cha, Hyun Hwangbo, Cheol Park, Hyesook Lee, Kyoung Seob Song, Jung-Hyun Shim, Jeong Sook Noh, Heui-Soo Kim, Bae-Jin Lee, Suhkmann Kim, Gi-Young Kim, You-Jin Jeon, Yung Hyun Choi

**Affiliations:** 1 Anti-Aging Research Center, Dong-eui University, Busan 47340, Republic of Korea; 2 Department of Biochemistry, Dong-eui University College of Korean Medicine, Busan 47227, Republic of Korea; 3 Department of Parasitology and Genetics, College of Medicine, Kosin University, Busan 49104, Republic of Korea; 4 Korea Nanobiotechnology Center, Pusan National University, Busan 46241, Republic of Korea; 5 Division of Basic Sciences, College of Liberal Studies, Dong-eui University, Busan 47340, Republic of Korea; 6 Department of Medical Life Science, College of Medicine, Kosin University, Busan 49104, Republic of Korea; 7 Department of Pharmacy, Mokpo National University, Jeonnam 58554, Republic of Korea; 8 Department of Food Science & Nutrition, Tongmyong University, Busan 48520, Republic of Korea; 9 Department of Biological Sciences, College of Natural Sciences, Pusan National University, Busan 46241, Republic of Korea; 10 Ocean Fisheries & Biology Center, Marine Bioprocess Co., Ltd., Busan 46048, Republic of Korea; 11 Center for Proteome Biophysics and Chemistry, Department of Chemistry, College of Natural Sciences, Institute for Functional Materials, Pusan National University, Busan 46241, Republic of Korea; 12 Department of Marine Life Science, Jeju National University, Jeju 63243, Republic of Korea

The journal retracts the article “Protection against Oxidative Stress-Induced Apoptosis by Fermented Sea Tangle (*Laminaria japonica* Aresch) in Osteoblastic MC3T3-E1 Cells through Activation of Nrf2 Signaling Pathway” [1] cited above.

Following publication, concerns were brought to the attention of the Editorial Office regarding the integrity of images presented in this publication [1].

Adhering to our standard procedure, an investigation was conducted by the Editorial Office and Editorial Board that identified indications of inappropriate editing and image duplication between Figures 4 and 7, presenting different experimental conditions. While the authors collaborated within this process, raw material meeting the journal’s Original Images Requirements could not be provided for Editorial Board Evaluation. Consequently, the Editorial Board has lost confidence in the reliability of the findings and has decided to retract this publication, as per MDPI’s retraction policy (https://www.mdpi.com/ethics#_bookmark30, accessed on 6 August 2025).

This retraction was approved by the Editor-in-Chief of the journal *Foods*.

The authors did not agree to this retraction.
